# Lack of health care professional’s awareness for management of celiac disease may contribute to the under diagnosis of celiac disease 

**Published:** 2019

**Authors:** Farnoush Barzegar, Mohammad Rostami-Nejad, Kamran Rostami, Soleyman Ahmadi, Hamid Mohaghegh Shalmani, Amir Sadeghi, Maryam Allahverdi Khani, David Aldulaimi, Mohammad Reza Zali

**Affiliations:** 1 *Student Research Committee, Gastroenterology and Liver Diseases Research Center, Shahid Beheshti University of Medical Sciences, Tehran, Iran*; 2 *Gastroenterology and Liver Diseases Research Center, Research Institute for Gastroenterology and Liver Diseases, Shahid Beheshti University of Medical Sciences, Tehran, Iran*; 3 *Department of Gastroenterology Palmerston North Hospital, New Zealand *; 4 *School of Medical Education, Shahid Beheshti University of Medical Sciences, Tehran, Iran*; 5 *Basic and Molecular Epidemiology of Gastrointestinal Disorders Research Center, Research Institute for Gastroenterology and Liver Diseases, Shahid Beheshti University of Medical Sciences, Tehran, Iran*; 6 *Faculty of Medicine, Najafabad Branch, Islamic Azad University, Najafabad, Iran*; 7 *Department of Gastroenterology, South Warwickshire Foundation Trust, UK*

**Keywords:** Instructional design, Celiac Disease, specialists, need assessment

## Abstract

**Aim:**

We assessed the knowledge of physicians regarding diagnosis and treatment of celiac disease (CD).

**Background:**

Specialists as the main therapist group of CD patients may play crucial role in the diagnosis and treatment of CD. Therefore, training and ensuring their capabilities is important.

**Methods:**

The population was specialists including Gastroenterologist, GI fellow, consultants, residents and general practitioners graduated in Medical Sciences Universities in Iran. The examination was the experts made and aimed to assess the educational needs of physicians and explore their failures in the diagnosis and treatment of CD with the key feature approach. Data was collected using a questionnaire that its validity and reliability was confirmed by experts (r = 91.6%). The total score was 150 with the classification of participants to the following categories: good (112- 150), intermediate (39-112) and weak ( ≤38).

**Results:**

Out of 300 participants, 197 questionnaires were returned (Response rate = 66%). The mean age of the participants was 42.67 years (SD = 7.9 years) with majority were male (63.6%). Average score of participates who had less than three year’s experience was significantly higher than others (P≤0.05). Only 12.1% and 9.8% of specialists have got the excellent score for diagnosis and treatment, respectively.

**Conclusion:**

It may conclude that specialists have had performance gap and around 90% needed training based on the principles of instructional design in order to improve their knowledge and skills to do and practice their assigned tasks. Therefore, development of training packages according to the principles of instructional design is suggested.

## Introduction

 Celiac disease (CD) is an autoimmune disorder, which leads to immune response and intestinal mucosal damage due to gluten consumption. The prevalence of CD in European countries is reported around 1% (1). Also the same prevalence is reported from the central Asia and Iran in people who are not at risk for the disease (2-4). The prevalence of the disease in Iran in general population is about 1% (5-7). The disease is common in childhood, adolescence and even adults, so that about 20% of patients are over 60 years old (8). Different signs and symptoms including typical (diarrhea, failure to thrive, anemia, weight loss) and atypical presentations (dyspepsia, fatigue, bone disease, liver enzyme elevation, and infertility) are reported in CD patients in children and adults (9, 10). Lack of timely treatment of celiac patients who have recently been diagnosed and have received gluten in their diet, can cause severe complications, including colon cancer, entropy associated with T cell lymphoma or persistent and severe diarrhea (11). 

Complications of CD are also associated with a lack of awareness in the area of diagnosis and treatment among health care professionals even with availability of sensitive and specific diagnostic tests such as immunoglobulin (Ig) A-tissue transglutaminase antibody. As the prevalence of atypical presentation in CD patient is more than typical and currently around 40% of diagnosed with CD are adults (12), the health care professionals may miss the timely diagnose of CD (13). Therefore, health care professionals must be conscious of CD complications/presentations in order to make an appropriate diagnosis. The knowledge and skills of internal health care professionals are not sufficient in comparison with the expectations of the health system, especially in the provision of services in the diagnosis and treatment of CD, and in addition to revision of the curriculum the training of health care professionals to raise the level of information and skills is required.

There is no document on the health care professional’s awareness of CD among Iranian population. Therefore, this study was designed to assess the knowledge of the Iranian health care professionals. In the recent study, we attempted to address the deficiencies of the health care professionals which can be solved by training. 

## Methods

The population studied in this study included Gastroenterologist, GI fellow, specialists, residents and general practitioners graduated from universities throughout the country who worked in private or public health centers in different cities.

In this study, health care professionals with a medical history of at least one year in the public or private sector were enrolled and considered as the target population of the study. Sampling method was selected by convenient and random sampling considering the variety and number of health care professionals participating in the gastrointestinal and liver general congresses during the comprehensive retraining program throughout the country, especially for health care professionals in Tehran. This study was designed with the aim of identifying functional defects of health care professionals in the diagnosis and treatment of CD, with a key feature approach. The formal and content validity of this study were evaluated by knowledgeable experts in the field of educational designing, diagnosis and treatment of CD. The reliability of the test was also tested by a pilot study and the internal consistency of the test items was confirmed with a correlation coefficient of 91.6%, which indicates a good reliability of the test (14). 

The data were collected using questionnaire. The test consisted of two parts: demographic information such as age, sex, place and year of graduation, city of activity, work experience, type of activity and 15 questions for assessing the knowledge and function of health care professionals for the diagnosis (11 questions), complications and treatment (4 questions) of CD, and identification of at risk people. Three hundred questionnaires were distributed. Those who were not willing to participate in research and failed to complete the questionnaire were excluded. Therefore, 103 people were excluded and, 197 questionnaires were returned. 

**Ethical consideration**

The study was approved by the Ethics Committee of Gastroenterology and Liver Diseases Research Center, Shahid Beheshti University of Medical Sciences, Tehran, Iran. 

**Statistical analysis**

Data were analyzed using statistical package for social sciences (SPSS) version 16 (SPSS Inc. Chicago, IL) for windows. The number and percentage of participants according to age, sex, place of graduation (universities in Tehran, universities in other cities), graduation year, city of activity (Tehran, other cities), work experience (Less than three years, between three and five years, between five and ten years, more than ten years) and the type of activity (private, public, both) was determined using the cross tab table. The number and percentage of correct answers to the questionnaire were determined and according to the 150 total score, the excellent group was classified if they were acquired 150-113 points, good: 112-79 points, intermediate: 39- 78 and weak: less than 38 points. Of the 150 scores, 110 scores were attributed to the diagnostic section and 40 scores were allocated for treatment. The statistical tests n and m Chi-square and Oneway ANOVA were used. A p-value <0.05 was considered as significant. 

## Results

Out of 300 participants, 197 questionnaires including 43.1% female and 56.9% male with the mean age of 36.83±10.370 years were completely returned. The majority of participants were 36-50 years old (91/46.2%). Most of the study population was GP (33.5%), followed by specialists (28.9%) and GI (17.3%) and mainly employed in governmental hospitals (45.7%). The demographic data of the study population are shown in Table 1. 

**Table 1:** Characteristics of the study participants

Characteristics		Number (%)
Sex	Male	85 (43.1)
	Females	112 (56.9)
Age group	0-35	86 (43.7)
	36-50	91 (46.2)
	51-100	19 (9.6)
Degree	GP	66 (33.5)
	Resident	17 (8.6)
	Specialist	57 (28.9)
	GI Fellow	23 (11.7)
	GI	34(17.3)
Type of Hospital	Private	26 (13.2)
	public	90 (45.7)
	Both	49 (24.9)
	Not employed	13 (6.6)
	Not mentioned	19 (9.6)

**Table 2:** The knowledge of health care professionals regarding diagnosis of celiac disease

		Weak (< 35) n(%)	Intermediate (36-112) n(%)	Good (113-150) n(%)	P Value
Sex	Male	13 (48.1)	65 (51.2)	34 (79.1)	0.004
	Female	14 (51.9)	62 (48.8)	9 (20.9)	
Degree	GP	10 (37)	51 (40.2)	5 (11.6)	0.0001
	Resident	5 (18.5)	11 (8.7)	1 (2.3)	
	Specialists	7 (25.9)	42 (33.1)	8 (18.6)	
	GI Fellow	4 (14.8)	11 (8.7)	8 (18.6)	
	GE	1 (3.7)	12 (9.4)	21 (48.8)	
Work experience	1 Year	2 (7.4)	4 (3.1)	0	0.0001
	3 Year	10 (37)	58 (45.7)	3 (7)	
	4-5 Year	4 (14.8)	19 (15)	5 (11.6)	
	6-9 Year	4 (14.8)	13 (10.2)	4 (9.3)	
	10-20 Year	7 (25.9)	33 (26)	31 (72.1)	
Age group	>35	13 (48.1)	67 (52.8)	6 (14)	0.0001
	36-50	12 (44.4)	49 (38.6)	30 (69.8)	
	51-100	2 (7.4)	11 (8.7)	7 (16.3)	

**Table 3:** The knowledge of health care professionals regarding the treatment of celiac disease

		Weak (< 35) n(%)	Intermediate (36-112) n(%)	Good (113-150) n(%)	P Value
Sex	Male	16 (61.5)	57 (53.8)	39 (60)	0.63
	Female	10 (38.5)	49 (46.2)	26 (40)	
Degree	GP	5(19.2)	40 (37.7)	21 (32.3)	0.0001
	Resident	4 (15.4)	10 (9.4)	3 (4.6)	
	Specialists	14 (53.8)	33 (31.1)	10 (15.4)	
	GI Fellow	2 (7.7)	11 (10.4)	10 (15.4)	
	GE	1 (3.8)	12 (11.3)	21 (32.3)	
Work experience	1 Year	1 (3.8)	3 (2.8)	2 (3.1)	0.002
	3 Year	10 (38.5)	40 (37.7)	21 (32.3)	
	4-5 Year	6 (23.1)	20 (18.9)	2 (3.1)	
	6-9 Year	6 (23.1)	10 (9.4)	5 (7.7)	
	10-20 Year	3 (11.5)	33 (31.1)	35 (53.8)	
Age group	>35	7 (26.9)	56 (52.8)	23 (35.4)	0.08
	36-50	17 (65.4)	40 (37.7)	34 (52.3)	
	51-100	2 (7.7)	10 (9.4)	8 (12.3)	

**Table 4:** Score obtained by the health care professionals participating in this study

	degree				
	GP	resident	specialist	fellow	GI
Weak	8 (33.3%)	3 (12.5%)	10 (41.7%)	2 (8.3%)	1 (4.2%)
Intermediate	51 (40.8%)	14 (11.2%)	41 (32.8%)	11 (8.8%)	8 (6.4%)
Good	7 (14.6%)	0	6 (12.5%)	10 (20.8%)	25 (52.1%)

The total score of knowledge of study population regarding the diagnosis and treatment was as follow: weak (12.2%), good (24.4%) and intermediate (63.5%), but separately the highest score regarding the diagnosis and treatment was belonged to the intermediate score, respectively (for diagnosis (127(64.5%)); treatment (106(53.8%)).

In general, there was a significant relationship between sex, age, degree and work experiences with respond to questions. The results showed that the 69.8% of participants in 36-50 years old group has achieved a score ‘good’ for diagnosis compared to other age groups and these differences were statistically significant (p=0.001) while they were not statistically significant for treatment score (p=0.08). Regarding the sex, the results indicated that, for diagnosis and treatment, the highest score was belonged to the intermediate score and this differences was statistically significant only for diagnosis (p=0.004 vs p=0.63). According to the work experience, the participants divided into 5 groups and results showed that those with work experience between 10 and 20 years had significantly good knowledge for diagnosis (p=0.0001) and treatment (p=0.002) compared with others. There are detailed reports on the response to treatment and diagnoses are presented in Tables 2 and 3.

The physicians were categorized into Gastroenterologist, GI fellow, specialists, residents and general practitioners. According to the questions related to the diagnosis and treatment of CD, the average score obtained by the physicians participating in this study were 58.03±19.24 and 23.46±8.84, respectively. 41.7% of specialists, 40.8% of GP and 52.1% of Gastroenterologist had weak, intermediate and good knowledge for diagnosis and treatment of CD, respectively. This difference between the studied groups was statistically significant (p=0.0001) (table 4).

## Discussion

Delay in diagnosis of CD can further lead to growth failure and delayed puberty in young children and atypical presentation in adults. The result of this study confirmed the lack of health care professional knowledge of CD, so that this poor knowledge may result in delay in diagnosis and suitable treatment. Early diagnosis by reducing the costs of medical care, as undiagnosed patients utilize several health care services, has significant financial impact on the patient’s life (15).

The findings of the research showed that health care professionals, despite the motivation, suffered the lack of sufficient knowledge and proper function in the diagnosis and treatment of CD. Only 24.4% of participants obtained an excellent score. The mean score with a minimum score of 0 and a maximum score of 150 points, for diagnosis was 58.03 ± 19.2 and for treatment was 23.46± 8.8. Considering the results of the study showed the gap between the favorable situation and the current state of health care professionals in the diagnosis and treatment of this disease, it was demonstrated that they had functional impairment due to knowledge. Accordingly, health care professionals need educational training in the diagnosis and treatment of CD. So according to the score, to solve this problem the design of the educational package seems necessary. 

Our results confirmed that the knowledge of CD among the Gastroenterologist, GI fellow was better, and this may because of their higher experiments in dealing with the patients. Specialist followed by general practitioners had weak level of knowledge. In contrast to our study, Assiri *et al.* (10) concluded that senior physicians like consultant had fared poorly compared to the other studied groups. Also in contrast to their findings, we showed that physicians with a work history of more than 10 years had better knowledge and better performance than other people in the diagnosis and treatment of CD. In a study by Zipers and his colleagues in the United States in 2005, they have analyzed physicians' awareness through a questionnaire of 2,440 patients. The results showed that CD was diagnosed by 11% of family physicians and internists and 65% by gastroenterologists (16). The study concluded that the lacked of family physicians and internists’ awareness of the onset of the symptoms of illness and related diseases is principal and they need to increase awareness of CD. 

As a gastroenterologists obtained higher knowledge score of CD (52.1%) compared to the other studies groups, we believe that it is because of their ability and experience to diagnose the patients with very mild or typical presentations, and the primary care physicians may not recognize them.

In conclusion, it was found that other than gastroenterologist, the participants in this study do not have the knowledge and the proper function in the diagnosis, treatment and prevention of complications of CD. The results proposed that health care professionals after completing their educational period are not well-versed in describing their duties in the field of diagnosis and treatment of CD. Therefore, the use of appropriate methods for assessing the educational needs of health care professionals and paying attention to the educational needs, followed by the development of a curriculum and the adaptation of educational content to the needs of this group, will enhance the quality and effectiveness of the curriculum and will ultimately lead to satisfaction of the patients. 
